# Mucosal Small Cell Neuroendocrine Carcinoma of the Gallbladder With Extraregional Lymph Node Metastasis: A Case Report

**DOI:** 10.7759/cureus.116386

**Published:** 2026-09-16

**Authors:** Akira Shinohara, Masakazu Hashimoto, Ryosuke Nakano, Keiichi Mori, Michiyo Kodama, Masahiro Serikawa, Tamito Sasaki, Katsunori Shinozaki, Takashi Nishisaka, Hideki Nakahara

**Affiliations:** 1 Department of Gastroenterological Surgery, Hiroshima Prefectural Hospital, Hiroshima, JPN; 2 Department of Gastroenterological and Transplant Surgery, Hiroshima University, Hiroshima, JPN; 3 Department of Diagnostic Pathology, Hiroshima Prefectural Hospital, Hiroshima, JPN; 4 Department of Clinical Oncology, Hiroshima Prefectural Hospital, Hiroshima, JPN; 5 Department of Gastroenterology, Hiroshima Prefectural Hospital, Hiroshima, JPN

**Keywords:** case report, chemotherapy, extraregional metastasis, gallbladder neuroendocrine carcinoma, small cell carcinoma

## Abstract

Gallbladder small cell neuroendocrine carcinoma (SCNEC) is a rare and highly aggressive malignancy, and an optimal treatment strategy has not been established. We report a case of mucosal gallbladder SCNEC with extraregional lymph node metastasis that responded well to chemotherapy. A 69-year-old woman presented with severe epigastric pain and inflammatory findings. Ultrasonography, endoscopic ultrasonography, and computed tomography revealed a 40 mm gallbladder mass and enlarged lymph nodes in the hepatoduodenal ligament. Because inflammation and pain could not be controlled by conservative treatment and endoscopic gallbladder drainage was unsuccessful, emergency laparoscopic cholecystectomy was performed. Histopathological examination revealed SCNEC with a Ki-67 index of 80%. Although continuous tumor invasion was limited to the mucosal layer and the tumor was classified as T1a, lymphatic invasion was present. Postoperative positron emission tomography and octreotide scintigraphy revealed metastases in the hepatoduodenal ligament and extraregional perisplenic artery lymph nodes. Further surgical resection was not performed, and chemotherapy with cisplatin and irinotecan was initiated. After six cycles, the hepatoduodenal ligament lymph node decreased from 31 mm to 13 mm, and the perisplenic artery lymph node disappeared, resulting in a partial response. No disease progression was observed during the 12-month postoperative follow-up period. This case suggests that gallbladder SCNEC may have metastatic potential even when the primary tumor is limited to the mucosal layer. Careful systemic staging is important, and platinum-based chemotherapy may be effective for metastatic disease.

## Introduction

According to the current World Health Organization classification, neuroendocrine neoplasms are broadly divided into well-differentiated neuroendocrine tumors and poorly differentiated neuroendocrine carcinomas. Neuroendocrine carcinomas are further classified into small cell and large cell types.

Small cell neuroendocrine carcinoma (SCNEC) of the gallbladder is classified as a small cell type of poorly differentiated neuroendocrine carcinoma arising in the gallbladder and is an extremely rare and aggressive malignancy, accounting for less than 1% of all gallbladder cancers [1,2]. Despite its rarity, it is associated with a poor prognosis, with an average survival of approximately eight months and a 0% five-year survival rate [3-5]. Histopathologically, gallbladder SCNEC is characterized by the sheet-like, nested, or trabecular proliferation of small tumor cells with scant cytoplasm and a high nuclear-to-cytoplasmic ratio. The tumor cells commonly exhibit finely granular chromatin, nuclear molding, numerous mitotic figures, apoptosis, and extensive necrosis. Immunohistochemically, the tumor cells are positive for neuroendocrine markers, including synaptophysin, chromogranin A, insulinoma-associated protein 1 (INSM1), and CD56, as well as cytokeratin, and generally show a high Ki-67 labeling index. Surgical resection is the preferred treatment for resectable cases; in advanced-stage gallbladder neuroendocrine carcinomas (GB-NECs), combining surgical resection with chemotherapy is superior to surgical resection alone, whereas chemotherapy is typically used in patients with unresectable tumors [6]. Chemotherapy regimens often follow the protocols used for small cell lung cancer [7], although the optimal drug combinations are still under investigation, and results from clinical trials are eagerly awaited. Here, we report a case of gallbladder SCNEC that, despite being confined to the mucosal layer, showed findings suggestive of both regional and extraregional lymph node metastases, with marked regression of the suspected metastatic lymph nodes after chemotherapy.

## Case presentation

A 69-year-old woman presented to a previous hospital with severe epigastric pain lasting 12 hours. Initial laboratory tests performed at the previous hospital revealed leukocytosis, and abdominal ultrasonography showed gallbladder debris; therefore, she was referred to our institution. On physical examination, spontaneous pain and tenderness were noted from the epigastric region to the right subcostal area, and Murphy's sign was positive. Laboratory tests on admission showed a C-reactive protein (CRP) level of 0.72 mg/dL and a white blood cell (WBC) count of 15,700/μL.

Abdominal ultrasonography and endoscopic ultrasonography (EUS) showed a well-circumscribed, round gallbladder mass measuring 43×34×30 mm with internal heterogeneity, and the No. 12 lymph node was enlarged to 25 mm. Contrast-enhanced computed tomography (CT) performed at our institution revealed gallbladder distension and mild gallbladder wall thickening, without areas of poor enhancement suggestive of necrosis. Early enhancement of the hepatic bed was also observed. A well-circumscribed, round mass measuring 40 mm was identified within the gallbladder. The mass had a heterogeneous internal structure, and no clear site of attachment was identified (Figure 1).

**Figure 1:**
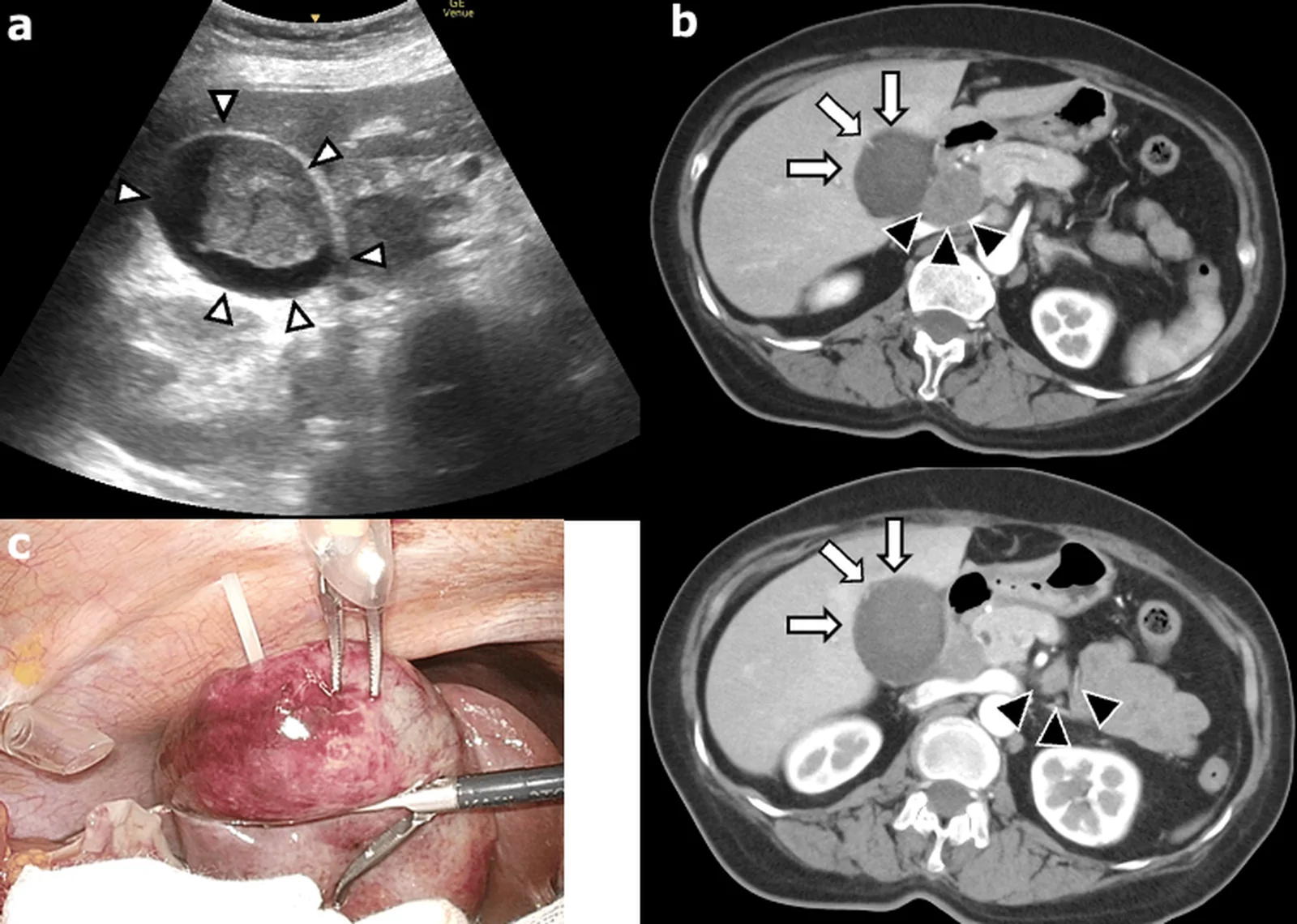
Ultrasonography, computed tomography, and intraoperative findings (a) Ultrasonography image showed a 40 mm hypoechoic mass in the neck of the gallbladder (△). No gallstones or gallbladder enlargement were observed. Lymph node enlargement was observed in the hepatic hilum. (b) Computed tomography revealed contrast enhancement irregularities in the liver surrounding the gallbladder, indicating cholecystitis (⇧). Additionally, a 40 mm mass was observed within the gallbladder, exhibiting slightly greater contrast enhancement than the surrounding tissue. Lymph node station 12 was enlarged, measuring 25 mm on the short axis, whereas lymph node station 11p measured 13 mm on the short axis (▲). (c) The patient underwent laparoscopic cholecystectomy, with an operative time of 132 minutes and an intraoperative blood loss of 43 mL. The gallbladder was tense and difficult to grasp with forceps, necessitating decompression through aspiration. The bile within the gallbladder appeared white. The resected gallbladder was sent for pathological examination.

Lymph node swelling was observed in the hepatoduodenal ligament and along the splenic artery. A gallbladder tumor was suspected, and gallbladder adenocarcinoma and neuroendocrine tumor were considered in the differential diagnosis. Because the tumor was a relatively large, well-circumscribed intraluminal mass with limited direct invasion beyond the gallbladder wall but prominent lymph node metastases, a neuroendocrine tumor was suspected rather than adenocarcinoma.

The clinical diagnosis was grade 1 acute cholecystitis associated with a gallbladder neoplasm with suspected lymph node metastasis.

After consultation with the gastroenterology department, we planned to control the patient's symptoms and inflammation with conservative/endoscopic management before performing further oncological evaluation. Endoscopic nasogallbladder drainage (ENGBD) was attempted; however, guidewire insertion into the cystic duct was unsuccessful, making ENGBD infeasible.

The next day, laboratory tests showed worsening inflammatory markers, with a CRP level of 12.8 mg/dL and a WBC count of 19,100/μL. Because the patient's abdominal pain persisted and sufficient improvement could not be achieved with conservative treatment, her condition was considered clinically uncontrolled. Emergency surgical intervention was therefore deemed necessary, and emergency laparoscopic cholecystectomy was performed.

The operative time was 132 minutes, and the estimated intraoperative blood loss was 43 mL. Intraoperatively, the gallbladder was erythematous and markedly distended, making surgical manipulation difficult; therefore, decompression was performed by puncture aspiration. Considering the risk of peritoneal dissemination, the procedure was carefully performed to avoid bile leakage into the abdominal cavity. Severe inflammation was present in the hepatoduodenal ligament, and the enlarged lymph nodes could not be identified intraoperatively (Figure 1).

Histopathological examination revealed extensive epithelial detachment, whereas the residual epithelium exhibited severe necrosis, degeneration, and neutrophil infiltration (Figure 2).

**Figure 2:**
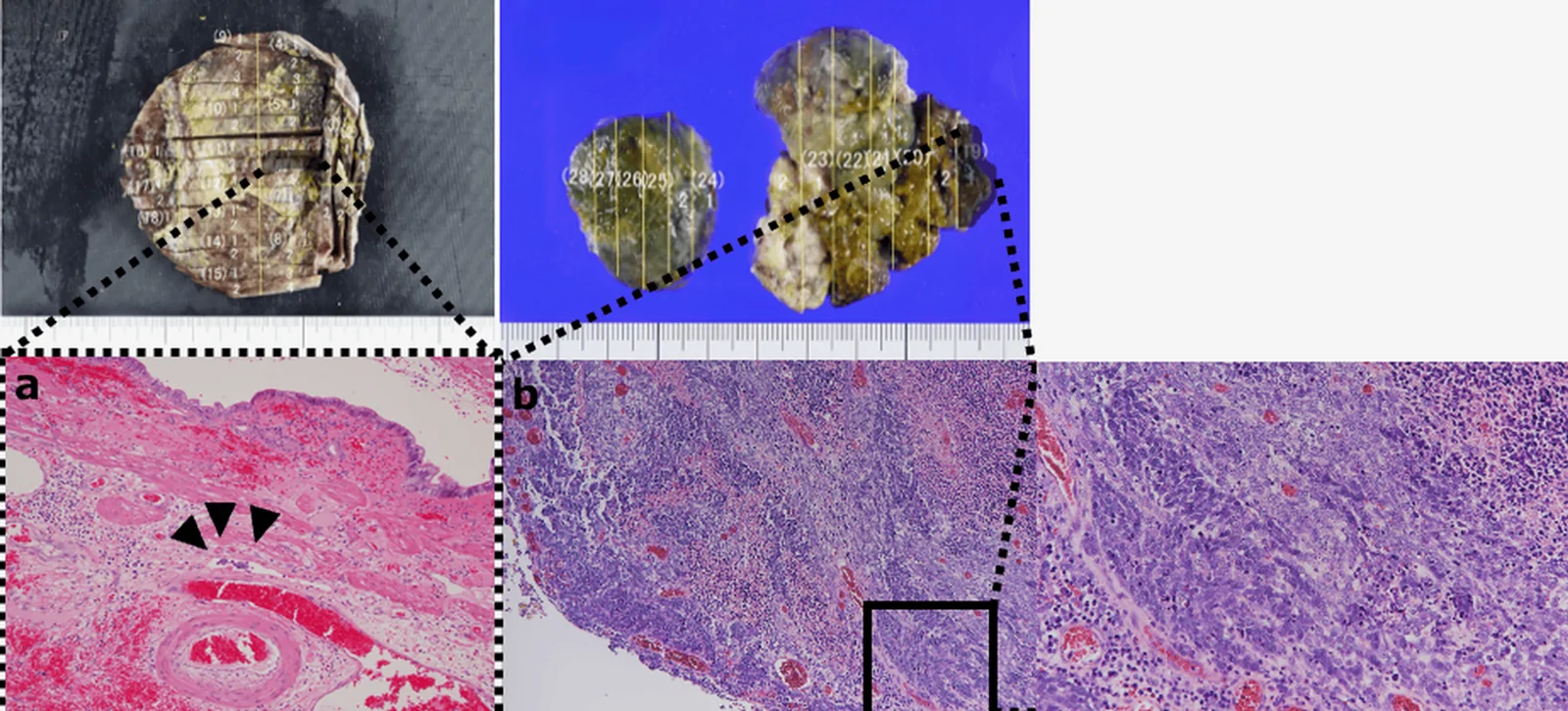
Histopathological examination Surgically resected specimen: The gallbladder and the mass were separate, and the mass was divided into 45 mm and 35 mm segments. (a) Continuous tumor invasion was observed up to the mucosal layer, with lymphatic invasion in the subserosal layer appearing as satellite-like lesions in the lymphatic vessels. (b) Hematoxylin and eosin staining showed small tumor cells with a high nucleus-to-cytoplasm (N/C) ratio proliferated in a trabecular pattern around blood vessels, with occasional mitotic figures.

Hematoxylin and eosin (H&E) staining revealed small cells with a high nuclear-to-cytoplasmic ratio proliferating in a trabecular pattern around vascular structures within the mucosal layer, with scattered mitotic figures and extensive necrosis (Figure 2). Tumor infiltration extended in a skip-like pattern into the lymphatic vessels of the subserosal layer (Figure 2). Immunohistochemical analysis showed positivity for synaptophysin, chromogranin A, cytokeratin, and CD56, with a Ki-67 proliferation index of 80% (Figure 3).

**Figure 3:**
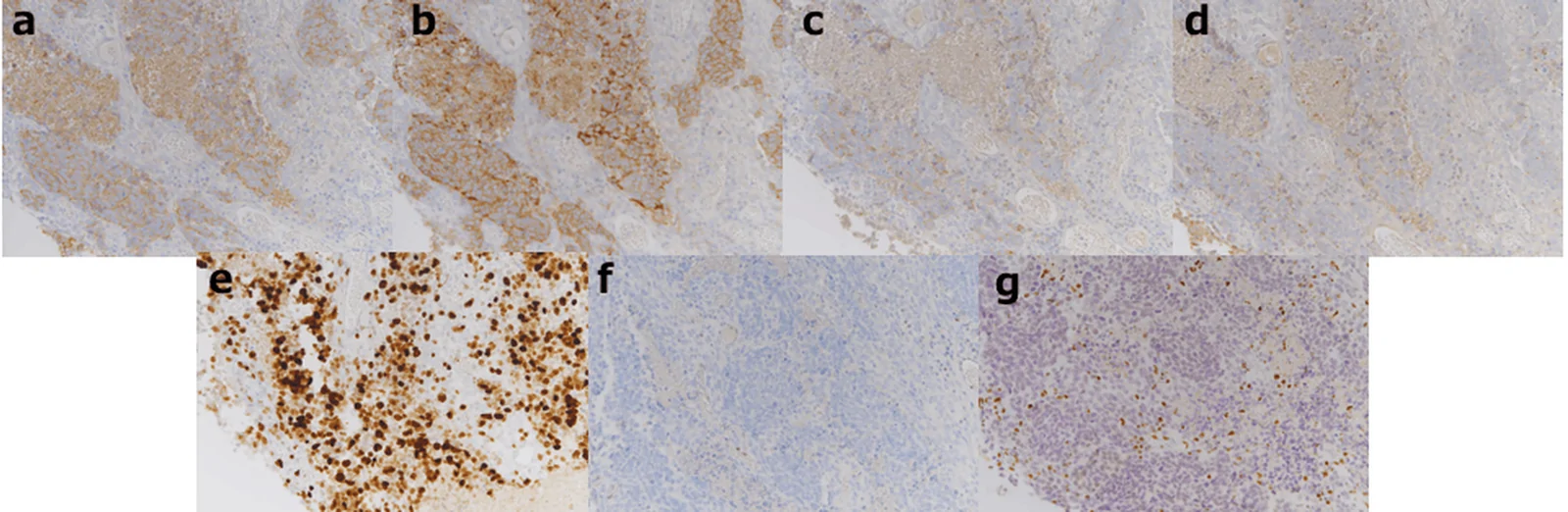
Immunohistochemical staining (original magnification, ×100 (a-g)) (a) Synaptophysin: tumor cells exhibited positive expression. (b) CD56: tumor cells exhibited positive expression. (c) Chromogranin A: tumor cells showed positive expression. (d) Cytokeratin: tumor cells exhibited positive expression. (e) Ki-67: tumor cells had a Ki-67 index of 80%. (f) p53: tumor cells exhibited negative expression (null pattern). (g) RB1: tumor cells exhibited negative expression.

Additionally, both p53 and RB1 staining were negative. These findings were consistent with a diagnosis of high-grade malignancy. Based on these findings, gallbladder SCNEC with lymphatic invasion, classified as T1a, was diagnosed.

Postoperative positron emission tomography-computed tomography (PET-CT) and octreotide scintigraphy demonstrated uptake not only in the hepatoduodenal ligament lymph node but also in the No. 11p lymph node along the proximal splenic artery, which was considered an extraregional lymph node. These findings raised strong suspicion of distant metastatic disease. Although histological examination of the No. 11p lymph node could have clarified whether this lesion represented metastasis from the gallbladder SCNEC or a different pathological process, we considered metastatic disease highly likely based on the location of the lesion in an extraregional nodal station and the overall clinical and imaging findings. Therefore, we considered that additional surgical exploration or resection would be unlikely to provide curative benefit and selected systemic chemotherapy rather than re-exploration (Figure 4).

**Figure 4:**
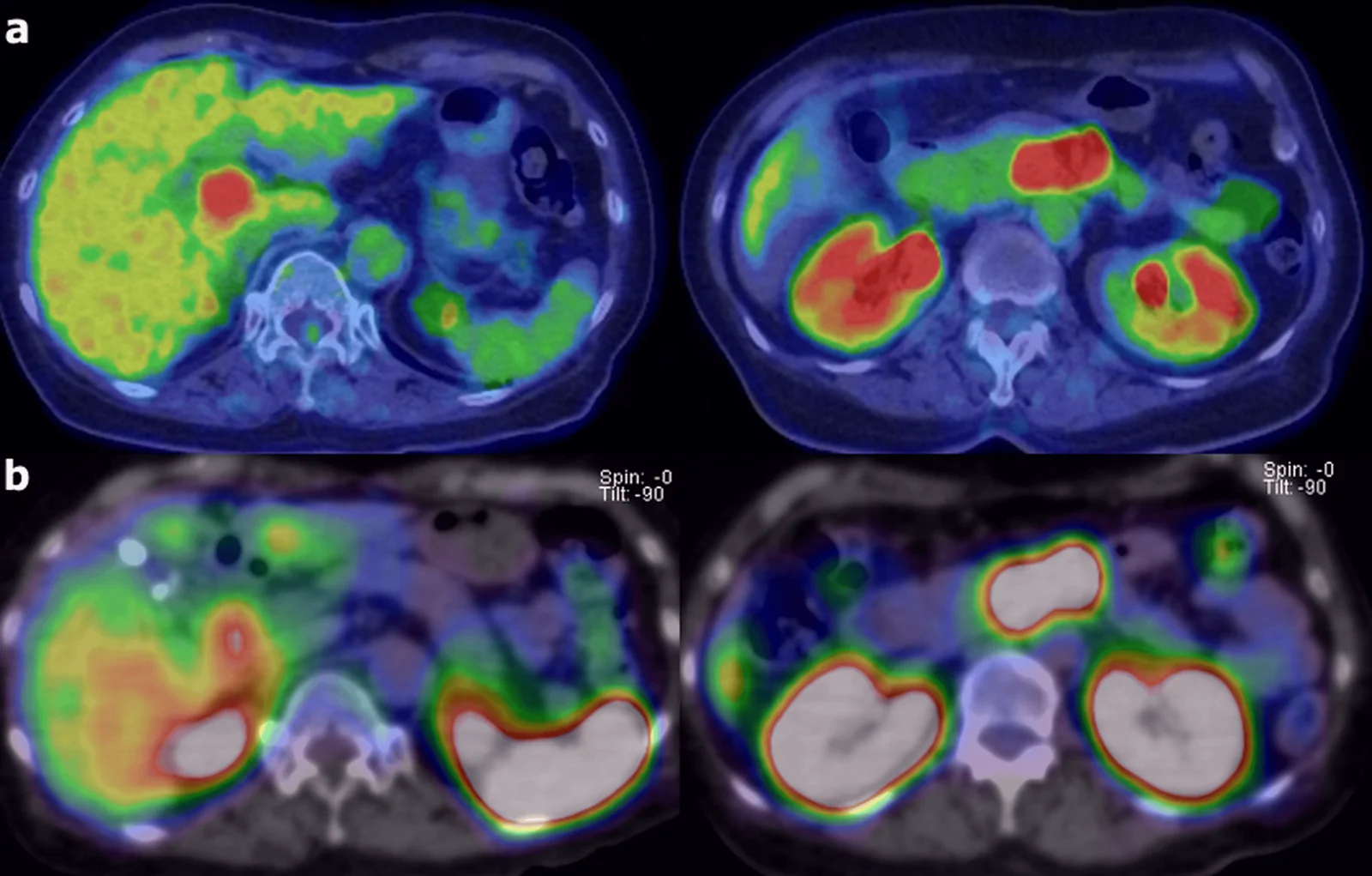
Positron emission tomography-computed tomography and octreotide scintigraphy Positron emission tomography-computed tomography (a) and octreotide scintigraphy (b) revealed tracer accumulation in lymph node station 12 and in extraregional lymph node station 11p.

Chemotherapy with cisplatin (CDDP) and irinotecan (CPT-11) was initiated. The initial regimen consisted of CDDP at 60 mg/m² on day 1 and CPT-11 at 60 mg/m² on days 1, 8, and 15, repeated every four weeks. During the first cycle, grade 3 diarrhea and grade 3 leukopenia developed according to the Common Terminology Criteria for Adverse Events, and the third dose of CPT-11 on day 15 was omitted. From the second cycle, the dose of CPT-11 was reduced to 80% of the initial dose (48 mg/m²), while CDDP was continued at 60 mg/m² on day 1 without dose reduction. During the third cycle, grade 3 febrile neutropenia developed, and the third dose of CPT-11 on day 15 was omitted. From the fourth through sixth cycles, CPT-11 was administered at 48 mg/m² on days 1, 8, and 15 without further dose reduction or omission. CDDP was administered at 60 mg/m² on day 1 throughout all six cycles. Before chemotherapy, the hepatoduodenal ligament lymph node (No. 12) and the proximal splenic artery lymph node (No. 11p) were enlarged to 31 mm and 13 mm, respectively. One month after the initiation of chemotherapy (after the completion of the first cycle), CT showed that the No. 12 and No. 11p lymph nodes measured 22 mm and 17 mm, respectively. Three months after the initiation of chemotherapy (after the completion of three cycles), the No. 12 lymph node had decreased in size to 15 mm, and the No. 11p lymph node had disappeared. Five months after the initiation of chemotherapy (during the sixth cycle), the No. 12 lymph node had further decreased in size to 13 mm, while the No. 11p lymph node remained undetectable (Figure 5).

**Figure 5:**
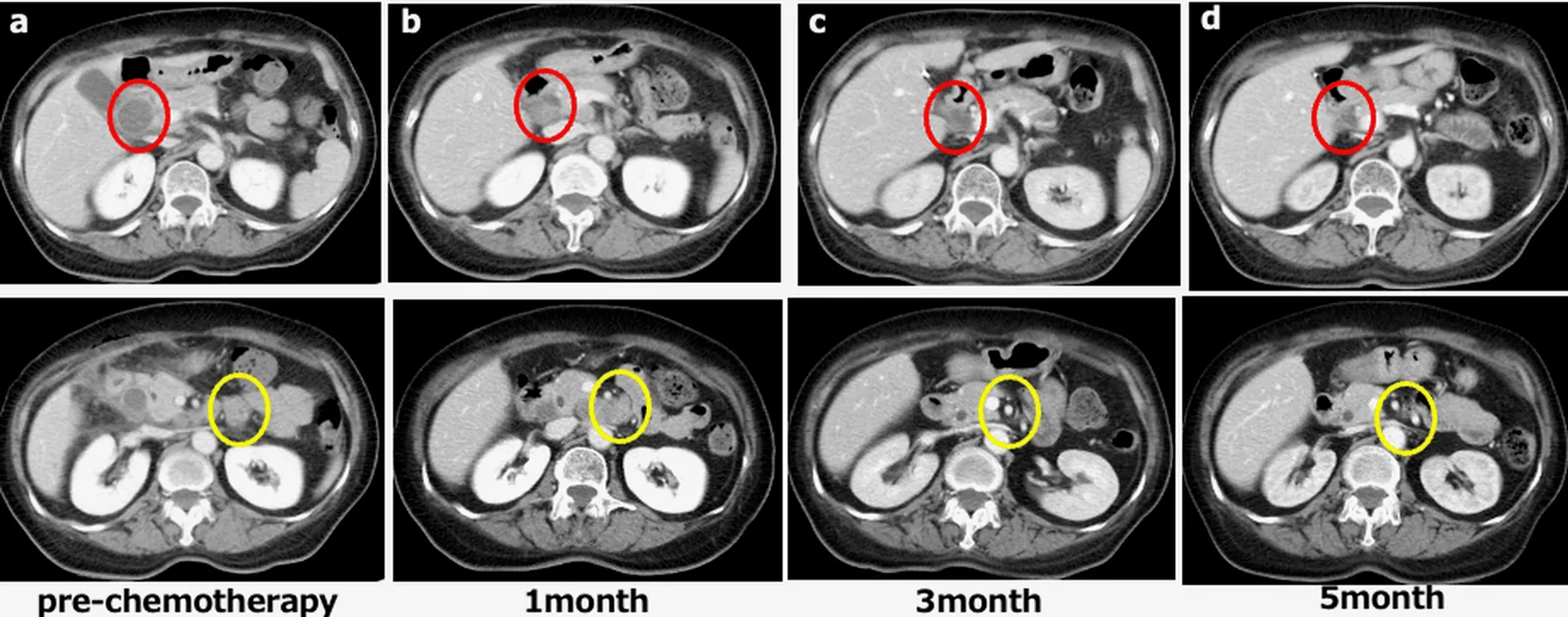
Serial computed tomography images obtained after the initiation of chemotherapy (a) Computed tomography image before chemotherapy. The No. 12 and No. 11p lymph nodes were enlarged to 31 mm and 13 mm, respectively. (b) Computed tomography image obtained one month after chemotherapy initiation. The No. 12 and No. 11p lymph nodes decreased in size to 22 mm and 17 mm, respectively. (c) Computed tomography image obtained three months after chemotherapy initiation. The No. 12 lymph node decreased in size to 15 mm, and the No. 11p lymph node disappeared. (d) Computed tomography image obtained five months after chemotherapy initiation. The No. 12 lymph node decreased in size to 13 mm, while the No. 11p lymph node remained undetectable. The metastatic lymph nodes showed marked regression after chemotherapy, and no disease progression was observed during follow-up.

After six cycles, the neuron-specific enolase (NSE) level decreased. Tumor response was assessed by CT after the first, fourth, and sixth cycles of chemotherapy. After the sixth cycle, the patient was assessed as having a partial response (PR), which represented the best overall response (Figure 5). Adverse events were evaluated according to the Common Terminology Criteria for Adverse Events, and tumor response was assessed according to the Response Evaluation Criteria in Solid Tumors (RECIST), Version 1.1.

## Discussion

Our case was a GB-NEC with suspected lymph node metastasis, although tumor invasion was limited to the mucosal layer. In addition, the suspected extraregional lymph node metastases responded well to chemotherapy, as shown by radiological regression.

According to the World Health Organization classification of digestive system tumors, neuroendocrine tumors are classified into three groups based on histological differentiation: well-differentiated neuroendocrine tumors, poorly differentiated neuroendocrine carcinomas, and mixed neuroendocrine tumors. Well-differentiated neuroendocrine tumors are further classified into G1, G2, and G3 based on the mitotic count and Ki-67 index [8].

SCNEC is difficult to diagnose by clinical findings and imaging alone, including CT, magnetic resonance imaging (MRI), and ultrasonography, and diagnosis is confirmed by histopathological examination [9]. Immunohistochemical staining is essential to confirm neuroendocrine differentiation. Common markers include synaptophysin, chromogranin A, and CD56. A high Ki-67 index usually indicates aggressive behavior of SCNEC [10], as seen in our case.

In our case, the tumor was 40 mm in size, and the Ki-67 index was high, at 80%. Therefore, the pathologists carefully evaluated the diagnosis of T1a (m). Tumor cells were found in the lymphatic vessels of the subserosal layer in a skip lesion-like pattern. However, the depth of invasion is evaluated based on the deepest point of continuous tumor invasion. In our case, the deepest continuous lesion was limited to the mucosal epithelium. Therefore, the tumor was diagnosed as T1a. Although extensive epithelial detachment and necrosis were observed in the gallbladder epithelium, only the epithelial layer had sloughed off, and the deeper layers were preserved. In addition, tumor cells were absent from most areas of the lamina propria and muscular layer, but were found in the residual mucosal epithelium. Based on these pathological findings, the tumor was classified as T1a. However, because the specimen quality was not fully preserved due to severe inflammation, the possibility of understaging cannot be completely excluded.

In our case, acute cholecystitis was initially suspected. However, a gallbladder tumor and enlarged surrounding lymph nodes were also observed. Therefore, we considered the possibility of an atypical gallbladder tumor. We planned to control the inflammation, perform further examinations, and provide appropriate treatment. Before further oncological evaluation, we planned ENGBD to decompress the gallbladder and control inflammation. Percutaneous transhepatic gallbladder drainage (PTGBD) was avoided because of the risk of tumor dissemination. However, the guidewire could not be inserted into the cystic duct, and ENGBD could not be performed. The next day, the patient's symptoms persisted, and laboratory tests showed worsening inflammatory markers. Therefore, emergency laparoscopic cholecystectomy was performed mainly to control the symptoms and inflammation, not for curative treatment. In general, CT, MRI, PET-CT, and octreotide scintigraphy are used to evaluate tumor characteristics, disease stage, and distant metastasis. If regional lymph nodes are enlarged, EUS-guided biopsy can be performed. After that, systemic chemotherapy followed by planned radical surgery can also be considered. Endoscopic ultrasound-guided fine-needle aspiration (EUS-FNA) has been reported to have high diagnostic performance [11].

Small cell-type GB-NEC is difficult to diagnose. Therefore, more than 90% of patients have distant or regional lymph node metastases at diagnosis [6]. Many patients with GB-NEC have advanced disease at diagnosis. The lymph nodes, liver, and lungs have been reported as common metastatic sites. In a study of 84 resected GB-NET cases, only seven cases (8.3%) were classified as T1, whereas 53 cases (63.1%) were classified as stage 3 or 4. The overall survival was 16.8 months [12]. Previous reports have shown the aggressive clinical course and poor prognosis of small cell carcinoma of the gallbladder. Adachi et al. [13] reviewed previous cases of gallbladder small cell carcinoma and showed that many patients had advanced disease and poor outcomes.

We performed a literature search on PubMed using the terms "gallbladder", "neuroendocrine tumor", and "neuroendocrine carcinoma", covering studies from 2000 to 2024. Among 347 reported cases of gallbladder neuroendocrine carcinoma, 65 were classified as pure small cell GB-NEC based on detailed pathological evaluation. After excluding 14 cases with unknown depth of invasion, 13 cases were classified as T2, 32 as T3 or T4, and six as T1 [14-19] (Table 1).

**Table 1:** T1 cases of gallbladder neuroendocrine carcinoma LN: lymph node; NA: not available; m: mucosa; mp: muscularis propria

Author	Age	Sex	Tumor size	Depth of invasion	Regional lymph node metastasis	Extraregional lymph node metastasis	Distant metastasis	Postoperative recurrence	Treatment strategy	Outcome
Majumder and Dhakane [14]	58	F	25 mm	mp	NA	NA	NA	NA	Laparoscopic cholecystectomy	NA
Wang et al. [15]	56	F	20 mm	mp	＋	NA	-	NA	Cholecystectomy + chemotherapy	15 months (dead)
Liu et al. [16]	69	F	19 mm	mp	+	-	-	-	Laparoscopic cholecystectomy	29 months (alive)
Maitra et al. [17]	71	M	NA	m	-	NA	-	NA	Cholecystectomy	3 months (dead)
Lane et al. [18]	67	M	NA	mp	-	-	-	Liver, LN	Extended cholecystectomy	11 months (dead)
Aiello et al. [19]	55	F	15 mm	mp	+	-	-	-	Laparoscopic cholecystectomy + chemotherapy	18 months (alive)
Current study	69	F	40 mm	m	+	+	LN	LN	Laparoscopic cholecystectomy + chemotherapy	12 months (alive)

GB-NEC is known to be highly aggressive and associated with a poor prognosis, even in relatively early-stage disease. However, the important feature of our case is that suspected extraregional lymph node metastasis was detected, although the primary tumor was classified as T1a and was limited to the mucosal layer. Of the six previously reported T1 cases, regional lymph node metastasis was present in four of the six cases, and metastasis was identified before surgery in only one case. However, PET or octreotide scintigraphy was not performed in any of the six cases. Therefore, the possibility of insufficient staging cannot be excluded. Our case suggests that even superficially invasive GB-NEC may have metastatic potential. Therefore, careful staging using systemic imaging should be performed whenever possible.

In our case, postoperative PET-CT and octreotide scintigraphy showed uptake in the proximal splenic artery lymph node, which is an extraregional lymph node. Therefore, the lesion was considered a distant metastasis, and systemic chemotherapy was selected instead of additional surgical resection. Chemotherapy resulted in marked regression of the suspected lymph node metastasis. No disease progression was observed during the 12-month postoperative follow-up period. However, the follow-up period was relatively short. Therefore, this result should be interpreted as a favorable short-term response, not as evidence of long-term disease control. Careful long-term follow-up is required. In patients with advanced GB-NEC, surgery combined with chemotherapy provides better outcomes than surgery alone [20]. Moskal et al. reported that patients with GB-NEC who underwent only gallbladder resection had a median survival of 4-5 months, whereas those who also received chemotherapy survived for 13 months [6].

Currently, no standard chemotherapy regimen has been established specifically for GB-NEC. However, platinum-based chemotherapy regimens are commonly used. The European Neuroendocrine Tumor Society (ENETS) 2023 guidance [21] recommends platinum plus etoposide as the preferred first-line treatment for digestive neuroendocrine carcinoma. In contrast, irinotecan-based regimens are mainly considered later-line treatment options.

According to the Japanese Neuroendocrine Tumor Society (JNETS) guidelines [22], platinum-based chemotherapy regimens, including etoposide plus cisplatin (EP) and irinotecan plus cisplatin (IP), are treatment options for neuroendocrine carcinoma. The TOPIC-NEC trial (JCOG1213), reported by Morizane et al. [23], compared EP with IP in patients with advanced digestive neuroendocrine carcinoma. This trial showed no statistically significant difference in median overall survival between the two regimens. In addition, no significant differences were found in progression-free survival, response rate, disease control rate, or adverse events. In our case, the chemotherapy regimen was selected by the Department of Clinical Oncology at our institution. The IP regimen was selected because our institution has extensive clinical experience with this regimen. Treatment with CDDP and CPT-11 resulted in marked regression of the suspected metastatic lymph nodes on CT.

Regarding future treatment strategies for GB-NEC, immune checkpoint inhibitors have gained attention. Although data are limited, Chorath et al. reported that combination therapy with the PD-1 inhibitor nivolumab, the CTLA-4 inhibitor ipilimumab, and EP chemotherapy achieved symptom relief in a patient with GB-NET [24]. Similarly, Li et al. reported a reduction in lymph node and liver metastases using FOLFIRI chemotherapy, consisting of irinotecan, calcium folinate, and fluorouracil, combined with toripalimab [20].

Further studies are needed to evaluate the efficacy of chemotherapy combined with immune checkpoint inhibitors in GB-NEC.

## Conclusions

We report a rare case of mucosal SCNEC of the gallbladder with suspected extraregional lymph node metastasis. Although the primary tumor was diagnosed as being confined to the mucosal layer (T1a), extraregional lymph node metastasis was suspected at diagnosis. Platinum-based chemotherapy with CDDP and CPT-11 was associated with a favorable treatment response, including marked regression of the lymph nodes suspected of harboring metastases. Careful long-term follow-up is warranted.
